# Xyloidine, or Gun-Cotton

**Published:** 1847-01

**Authors:** 


### XYLOIDINE, OR GUN-COTTON.

This article is at present exciting the attention of the whole scientific world. As mentioned by our correspondent from Paris, it is prepared by subjecting cotton, for a short time, to the action of concentrated nitric acid. That prepared by distilling ten parts of dry nitrate of potash with six parts of sulphuric acid, and having the specific gravity of 1.4, is found to be the best; it is preferable to the strongest nitric acid, which has a specific gravity of 1.52. The cotton is to be steeped in this acid for two or three minutes, and afterwards washed in water until litmus is no longer reddened by the water. It may also be made by immersing cotton for two hours in a mixture of the nitric and sulphuric acids of commerce, but thus prepared it is said to be more liable to spontaneous combustion than when made by the other process.

The gun-cotton burns suddenly with a bright yellow flame, kindling at a temperature of 420°, and may be ignited by the electricity of a Leyden jar, percussion on an anvil, or the ordinary methods of inflaming gunpowder. It is resolved entirely into nitrogen and carbonic acid gases, and consequently leaves no solid matter behind. The suddenness with which it is deflagrated is evinced by the circumstance, that it may be burnt upon gunpowder without kindling it, as is the case with hydrogen and the fulminating compounds of mercury and silver. Its expulsive force appears to be greater than that of gunpowder, but whether it will ever take the place of that agent must depend upon a variety of circumstances. Notwithstanding the simplicity of the process by which it is prepared, it is said that it will be more expensive than gunpowder. Another objection urged against it, we know not with what truth, is, that the chemical action does not cease with the drying of the cotton, but that changes continue to go on by which its quality is gradually impaired. It only explodes with force when perfectly dry, and as cotton attracts the moisture of the atmosphere with much energy, it is difficult to avoid some degree of humidity. It is also objected to it, that it sometimes inflames spontaneously in drying, and that, consequently, it would not be safe to keep a quantity of it together; that it is subject to being ignited by a heated gun, or by driving the ram rod home incautiously, and that it is more apt than gunpowder to burst the gun, owing to the suddenness of its explosion. Experience alone can determine the force of these objections, and fix the utility of the article.

Of Germany it has been sung:

> "Mars owes to thee his thunder
> That shakes the battle field;"

and to her discoveries of the clock, the printing press, and gunpowder, the world generally has consented to allow her to add that of gun-cotton; but from some recent notices on the subject we see that France is preferring a claim to the honor of the discovery. Pelouze, in some experiments with nitric acid on the woody fibre, performed several years ago, ascertained that the qualities of the substance, whether paper, linen, or saw dust, were materially altered. This new principle, termed *xyloidine*, (from *xulon* wood, and *eidos* like), was found to burn rapidly, and often with explosive violence. The works of Turner and Graham on chemistry, published in 1842, allude to this effect of nitric acid on the ligneous fibre. Turner says, "In strong nitric acid saw dust dissolves; and on the addition of water, a white insoluble powder is deposited, which contains nitric acid, and explodes when heated." Graham is still more explicit in the subjoined extract:—"Nitric acid, in its highest state of concentration, exerts no violent action upon certain organic substances, such as lignin or woody fibre and starch, for a short time, but unites with them, and forms singular compounds. A proper acid for such experiments is procured with most certainty by distilling 100 parts of nitric acid with no more than 60 parts of the strongest oil of vitriol. If paper is soaked for one minute in such an acid, and afterwards washed with water, it is found to shrivel up a little, and become nearly as tough as parchment, and, when dried, to be remarkably inflammable, catching fire at so low a temperature as 356°, and burning without any nitrous odor, (Pelouze)." Am. Ed. p. 219.

Dumas, in his Traité de Chimie, mentions that these properties suggested to Pelouze the idea of employing paper or cloth, so treated, in the manufacture of cartridges for artillery; and he even went so far, at a late meeting of the Academy of Sciences, in Paris, as to assert that his countryman announced the plan of applying these substances to the very purpose for which Schönbein has taken out a patent. Whether or not the German chemist was acquainted with the researches of his neighbor, Pelouze, it appears clear to us, was the real inventor of the process for preparing the explosive cotton.

